# Particle Size Measurement and Detection of Bound Proteins of Non-Porous/Mesoporous Silica Microspheres by Single-Particle Inductively Coupled Plasma Mass Spectrometry

**DOI:** 10.3390/molecules29051086

**Published:** 2024-02-29

**Authors:** Shin-ichi Miyashita, Toshihiko Ogura, Shun-ichi Matsuura, Eriko Fukuda

**Affiliations:** 1 National Metrology Institute of Japan (NMIJ), National Institute of Advanced Industrial Science and Technology (AIST), 1-1-1 Umezono, Tsukuba 305-8563, Ibaraki, Japan; shinichi-miyashita@aist.go.jp; 2 Health and Medical Research Institute, National Institute of Advanced Industrial Science and Technology (AIST), 1-1-1 Higashi, Tsukuba 305-8566, Ibaraki, Japan; t-ogura@aist.go.jp; 3 Research Institute for Chemical Process Technology, National Institute of Advanced Industrial Science and Technology (AIST), 4-2-1 Nigatake, Miyagino-ku, Sendai 983-8551, Miyagi, Japan; matsuura-shunichi@aist.go.jp; 4 Cellular and Molecular Biotechnology Research Institute, National Institute of Advanced Industrial Science and Technology (AIST), 1-1-1 Higashi, Tsukuba 305-8565, Ibaraki, Japan

**Keywords:** silica, microsphere, single-particle ICP-MS, SEM, size measurement, iron-containing proteins

## Abstract

Single-particle inductively coupled plasma mass spectrometry (spICP-MS) has been used for particle size measurement of diverse types of individual nanoparticles and micrometer-sized carbon-based particles such as microplastics. However, its applicability to the measurement of micrometer-sized non-carbon-based particles such as silica (SiO_2_) particles is unclear. In this study, the applicability of spICP-MS to particle size measurement of non-porous/mesoporous SiO_2_ microspheres with a nominal diameter of 5.0 µm or smaller was investigated. Particle sizes of these microspheres were measured using both spICP-MS based on a conventional calibration approach using an ion standard solution and scanning electron microscopy as a reference technique, and the results were compared. The particle size distributions obtained using both techniques were in agreement within analytical uncertainty. The applicability of this technique to the detection of metal-containing protein-binding mesoporous SiO_2_ microspheres was also investigated. Bound iron (Fe)-containing proteins (i.e., lactoferrin and transferrin) of mesoporous SiO_2_ microspheres were detected using Fe as a presence marker for the proteins. Thus, spICP-MS is applicable to the particle size measurement of large-sized and non-porous/mesoporous SiO_2_ microspheres. It has considerable potential for element-based detection and qualification of bound proteins of mesoporous SiO_2_ microspheres in a variety of applications.

## 1. Introduction

Inorganic supports, such as silica (SiO_2_) microspheres, have become increasingly important for a variety of applications, including the isolation of nucleic acids [1], cell separation [2], immuno-based [3] and DNA-based assays [4], and photocatalysis [5]. They offer the combined benefits of a broad platform and the unique properties of a SiO_2_ substrate: flexible silanization chemistry, unique refractive index and density, low autofluorescence, low nonspecific binding of many biomolecules, hydrophilicity, and ease of handling. Furthermore, mesoporous SiO_2_ with a pore size of 2–50 nm can encapsulate compounds, such as anticancer agents, or biomolecules, such as antigen proteins, within its regularly structured pores and release them in vivo. Because of these properties, various studies have been conducted on the feasibility of their use as drug delivery vehicles [6,7,8] and vaccine carriers [9,10,11]. For medical applications involving biological administration, it is important to evaluate SiO_2_ microsphere aggregation and closely assess the uniform binding of compounds and biomolecules to individual microspheres. This process ensures a homogeneous and contamination-free particle population and increases the feasibility of stringent lot-by-lot quality control measures. However, despite the multifaceted potential of SiO_2_ microspheres, current evaluation methods for particle characterization are often limited to bulk approaches such as dynamic light scattering and X-ray diffraction.

A promising technique for addressing this limitation is single-particle inductively coupled plasma mass spectrometry (spICP-MS), which is widely employed to determine the size and number of various individual nanoparticles (NPs) [12,13,14,15,16]. This encompasses non-porous/mesoporous SiO_2_ nanospheres [17] and nanometer/micrometer-sized carbon-based particles, such as nanoplastics and microplastics [15,18,19,20,21,22]. The approach is based on the one-by-one introduction of particles into the ICP ion source, in which the particles are destroyed, and their contents are vaporized, atomized, and ionized. Every individual particle that reaches the ICP yields a burst of ions that can be detected by MS. This provides an advantageous set of features: (i) it only requires an exceedingly small amount of particulate sample (micrograms or even less) in the form of a dilute dispersion (e.g., in a few milliliters at a concentration of 10^5^ particles/mL); and (ii) the measurement and calculation are quick (it takes only a few minutes) and simple. The basis and applications of spICP-MS have been described in many studies, indicating that this technique is suitable for particle characterization [12,13,14,15,18]. However, its applicability to the measurement of micrometer-sized non-carbon-based particles, such as SiO_2_ particles, is unclear.

In this study, we investigated the applicability of spICP-MS to the particle size measurement of non-porous/mesoporous SiO_2_ microspheres by comparing the measurement results obtained using this technique with those obtained by scanning electron microscopy (SEM), which was used as a reference technique. Moreover, we investigated the applicability of this technique to the detection of metal-containing protein-binding mesoporous SiO_2_ microspheres as one of its potential applications.

## 2. Results and Discussion

### 2.1. Particle Size Measurement by spICP-MS and SEM

**Figure 1:**
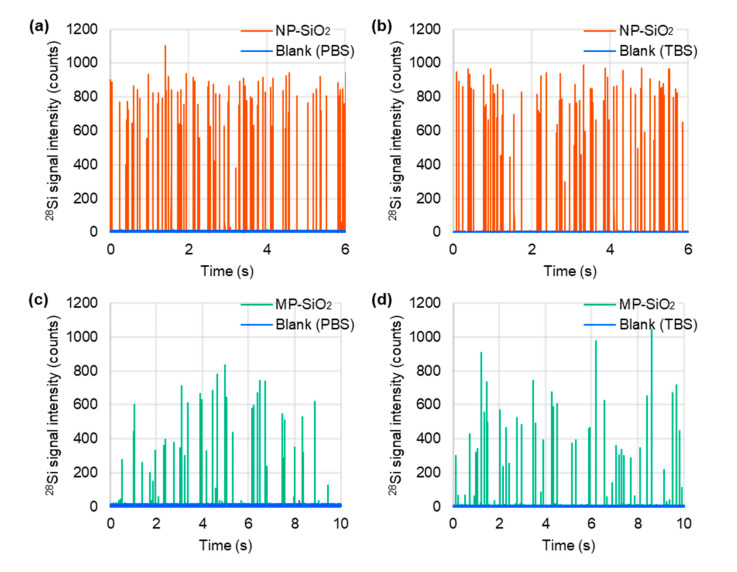
Representative time-resolved profiles for non-porous SiO_2_ microspheres (NP-SiO_2_) (**a**,**b**) and mesoporous SiO_2_ microspheres (MP-SiO_2_) (**c**,**d**) suspended in PBS/TBS, obtained by spICP-MS. A quadrupole ICP-MS instrument (Agilent 7700x ICP-MS) equipped with an ICP torch with an injector tube of diameter 1.5 mm, a conventional MicroMist nebulizer, and a Scott double-pass spray chamber cooled at 2 °C was used in combination with an externally assembled high-speed pulse signal processing system.

**Figure 2:**
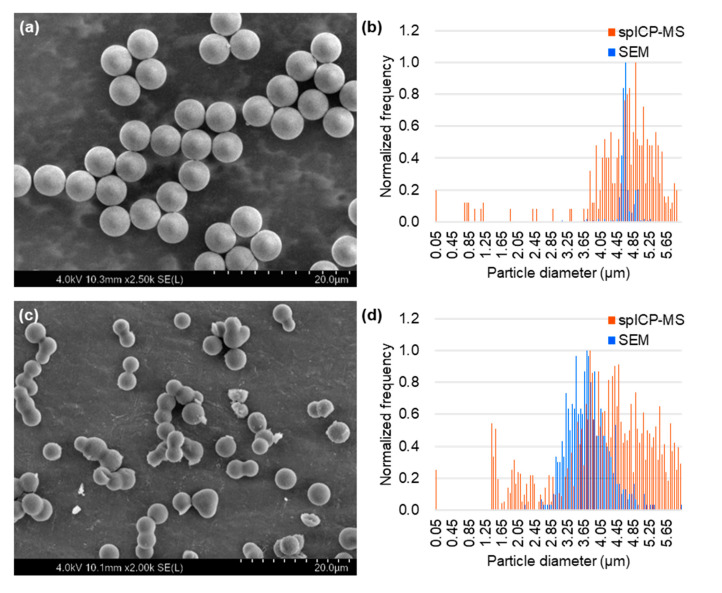
SEM images and particle size distributions of non-porous SiO_2_ microspheres (**a**,**b**) and mesoporous SiO_2_ microspheres (SBA24) (**c**,**d**) suspended in PBS, obtained from spICP-MS and SEM. For particle size measurement by spICP-MS, a quadrupole ICP-MS instrument (Agilent 7700x ICP-MS) equipped with an ICP torch with an injector tube of diameter 1.5 mm, a conventional MicroMist nebulizer, and a Scott double-pass spray chamber cooled at 2 °C was used in combination with an externally assembled high-speed pulse signal processing system. For particle size measurement by SEM, a FE-SEM instrument (SU5000) was used. Secondary electron images (1280 × 1020 pixels) were captured at 2000–2500× magnification with a scanning time of 20 s, a working distance of 7 mm, an EB acceleration voltage of 3–4 kV, and a current of 1–5 pA.

The particle size of the non-porous/mesoporous SiO_2_ microspheres was measured by (i) spICP-MS based on a conventional calibration approach using an ion standard solution and (ii) SEM as a reference technique, and the obtained results were compared. In the case of spICP-MS, the particle density ($\rho_{particle}$) value of 2.371 g/cm^3^ (average of triplicate measurements) measured using the gas pycnometry method was used for calculation. Representative time-resolved profiles of non-porous SiO_2_ microspheres and mesoporous SiO_2_ microspheres (SBA24) obtained by spICP-MS are shown in Figure 1. SEM images and particle size distributions of the non-porous/mesoporous SiO_2_ microspheres obtained from spICP-MS and SEM are shown in Figure 2. The SEM images of the non-porous/mesoporous SiO_2_ microspheres showed a spherical shape (Figure 2a,c) and the presence of some aggregates only in the mesoporous SiO_2_ microsphere suspension (Figure 2c). The particle size distributions of the non-porous/mesoporous SiO_2_ microspheres obtained using both techniques were in good agreement (Figure 2b,d). In spICP-MS, the average particle diameters and their standard deviations (SDs) of the non-porous/mesoporous SiO_2_ microspheres suspended in PBS were 4.97 ± 2.39 µm (*n* = 249) and 4.68 ± 2.40 µm (*n* = 839), respectively. They were almost in good agreement with the average particle diameters measured by SEM, 4.68 ± 0.19 µm (*n* = 400) for non-porous and 3.76 ± 0.49 µm (*n* = 569) for mesoporous SiO_2_ microspheres. For the non-porous SiO_2_ microspheres, the average particle diameter and their SDs obtained by spICP-MS also agreed well with those reported by Bangs Laboratories (i.e., 4.82 ± 0.38 µm). These results suggest that spICP-MS is applicable for the particle size measurement of large non-porous/mesoporous SiO_2_ microspheres.

Lee et al. [23] reported the typical lower-size detection limit (${LOD}_{size}$) range from approximately 10 to 40 nm for most monometallic particles, depending on the abundance of the analyte isotopes monitored. When working with alloys, oxides, or other compound or porous particles, the ${LOD}_{size}$ values usually increase because the analyte only constitutes a fraction of the particle mass [17]. The lower-size detection limits (${LOD}_{size,solid}$ and ${LOD}_{size,porous}$) calculated in this study were 241 nm for non-porous SiO_2_ microspheres and 441 nm for mesoporous SiO_2_ microspheres. Although the former value is close to the previously reported ${LOD}_{size,solid}$ value of 232 nm for commercially available non-porous (solid) SiO_2_ microspheres, the latter value is higher than the ${LOD}_{size,porous}$ value of the synthesized mesoporous Stöber SiO_2_ microspheres (292 nm) with an average porosity value of 50% [17]. This difference is due to the higher porosity (83.7%) of the mesoporous SiO_2_ microspheres used in this study, which results in higher ${LOD}_{size,porous}$ values according to Equation (4). However, the upper-size detection limits are less studied. Studies based on spICP-MS are significantly limited because the plasma tends to fully atomize and ionize particles during the transition (residence) time. This limit is also influenced by the dynamic capabilities of ICP-MS detection electronics, density, and boiling point of the compound [24]. Typical upper-size detection limits range from approximately 1 to 1.5 µm for solid SiO_2_ microspheres [25] and approximately 200 to 250 nm for solid gold (Au) particles [24,26]. In this study, the size of solid SiO_2_ microspheres was successfully measured to be 4.8 µm by spICP-MS, which experimentally shows that the spICP-MS-based particle size of SiO_2_ microspheres of approximately 5.0 µm is possible.

The ability to measure the particle size of non-porous/mesoporous SiO_2_ microspheres with the demonstrated detection limit may enable the evaluation of submicron particle aggregation states. As mentioned in the introduction, these microspheres are often used with adsorbed biomolecules, such as nucleic acids, proteins, and cells. These biomolecules can undergo denaturation or degradation over time and with temperature changes, potentially causing the aggregation of SiO_2_ microspheres. It might be difficult to microscopically distinguish between the proximity and aggregation of the microspheres. The integration of complementary data from both microscopy and spICP-MS enables a more comprehensive evaluation.

In this study, the overall porosity (83.7%) determined using a total pore volume ($V_{p}$) of 2.17 cm^3^/g for the mesoporous SiO_2_ microspheres was used to calculate the particle size. Porosity has a profound impact on particle chemistry because (i) it can make the particles permeable and (ii) an increase in the specific surface area increases the surface activity and adsorption of molecular species [27,28], thereby promoting various industrial and environmental science applications [29]. Recently, Kéri et al. [17] proposed a novel spICP-MS-based overall porosity determination method for nano- and sub-micron- particles (potentially, particles up to approximately 1–2 µm in size) with or without mesoporous pores. They demonstrated that the porosity of the synthesized mesoporous SiO_2_ NPs with an average diameter of approximately 400 nm (0.4 µm) could be determined by combining the information from spICP-MS (i.e., signal intensities from individual particles) with that from other NP characterization techniques (i.e., particle diameter or volume). The accuracy and precision of this method are comparable to those of other methods, such as small-angle X-ray scattering (SAXS), gas adsorption, and transmission electron microscopy. The overall porosity can also be used to calculate the density of the particles if the bulk density is known, which is not easy to determine because of the small amount of sample. The porosity of the mesoporous SiO_2_ microspheres calculated according to Equation (3) (using the spICP-MS data and the average particle diameter measured by SEM) was 68.4 ± 23.0% (*n* = 745), agreeing well with that separately calculated using Equation (5) (83.7%). According to our findings, the proposed spICP-MS-based porosity/density determination method is applicable to single micrometer-sized particles if they can be fully decomposed by plasma and their diameter and density are known. For example, the frequently used SAXS method requires a dry powder sample (tens of milligrams) and knowledge of the particle density, which may not be known for newly synthesized complex particles. However, the spICP-MS-based method requires a considerably smaller amount of sample (micrograms or less), which is a significant advantage when the sample quantity is limited. This method offers an additional benefit by automatically including both open (connected and permeable) and closed pores in the calculation.

### 2.2. Detection of Fe-Containing Protein-Binding Mesoporous SiO_2_ Microspheres by spICP-MS

**Figure 3:**
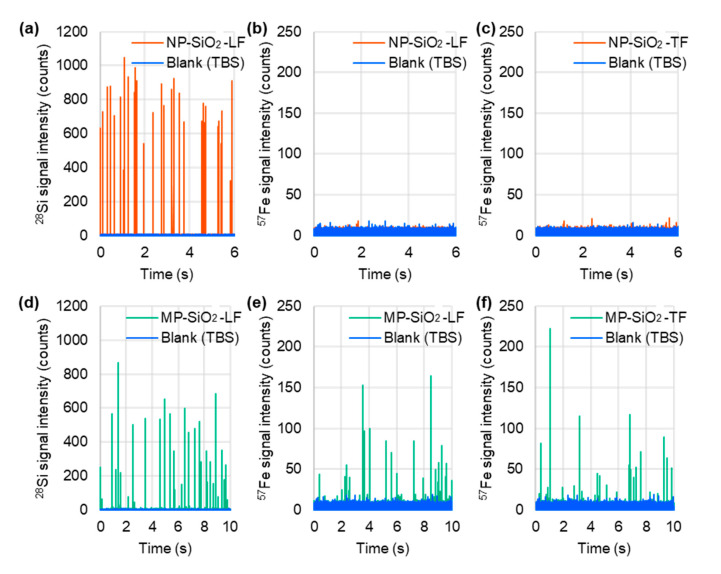
Representative time-resolved profiles for LF- and TF-binding non-porous SiO_2_ microspheres (NP-SiO_2_) (**a**–**c**) and mesoporous SiO_2_ microspheres (MP-SiO_2_) (**d**–**f**) suspended in TBS, obtained by spICP-MS while monitoring ^28^Si (**a**,**d**) and ^57^Fe (**b**,**c**,**e**,**f**) individually. A quadrupole ICP-MS instrument (Agilent 7700x ICP-MS) equipped with an ICP torch with an injector tube of diameter 1.5 mm, a conventional MicroMist nebulizer, and a Scott double-pass spray chamber cooled at 2 °C was used in combination with an externally assembled high-speed pulse signal processing system.

The applicability of spICP-MS to the detection of metal-containing protein-binding mesoporous SiO_2_ microspheres was investigated. The representative time-resolved profiles of the LF- and TF-bound mesoporous SiO_2_ microspheres obtained by spICP-MS are shown in Figure 3. LF and TF bound to the mesoporous SiO_2_ microspheres were detected using Fe as a marker for the presence of proteins. In contrast, little to no binding was observed in non-porous SiO_2_ microspheres. This observation suggests differences in the protein binding capacities between mesoporous and non-porous SiO_2_ microspheres. The results indicate that spICP-MS has considerable potential for element-based detection and qualification of bound proteins of mesoporous SiO_2_ microspheres in a variety of applications.

In this study, we detected the binding of LF and TF to mesoporous SiO_2_ microspheres by identifying Fe. LF is present in breast milk, supplying essential iron to newborns, while TF, found in the plasma, plays a role in transporting iron in the blood. Other Fe-binding proteins include heme proteins, such as hemoglobins and myoglobins, Fe storage proteins such as ferritin, and transcription factors that sense Fe levels. Thus, Fe-binding proteins play essential roles in biological processes. The binding of these proteins to mesoporous SiO_2_ microspheres is promising for enhancing the heat resistance of proteins, inducing immune responses for antibody generation in animals, and other potential applications. However, the number of Fe-containing proteins is limited. In addition to Fe, proteins contain elements such as sulfur, phosphorus, and carbon. Sulfur is a constituent of amino acids, such as cysteine and methionine, contributing to the formation of disulfide bonds and the overall protein structure. Phosphorus is integral to phosphorylation events and plays a crucial role in post-translational modifications of proteins. Carbon, present in all amino acids, is fundamental to the protein backbone. To broaden the ability to detect a wider range of proteins, future studies should focus on expanding the scope to analyze elements such as sulfur, phosphorus, and carbon.

When mesoporous SiO_2_ microspheres are used as carriers for drug delivery or as immune adjuvants, various molecules are adsorbed onto these microspheres depending on their purpose. These include proteins, peptides, nucleic acids, glycans, small-molecule drugs, polymers, lipids, and other molecules. Bulk assessments of molecules binding to these microspheres are feasible; however, the evaluation of each individual microsphere is limited. Although observation by labeling with fluorescent dyes or similar methods is possible, such labeling may alter the intrinsic behavior of molecules. The detection of proteins on the particles achieved in this study was performed with element selectivity, enabling the assessment of the presence of unlabeled proteins on a single-particle basis. It is also possible to distinguish between proteins, nucleic acids, lipids, and other components. This method is expected to provide valuable insights into the molecular biology and medical applications of mesoporous SiO_2_ microspheres. In the future, this achievement is expected to evolve into a promising method for assessing the homogeneity of prepared samples and evaluating the changes in the state of samples stored for a long time.

## 3. Materials and Methods

### 3.1. Materials

Non-porous/mesoporous SiO_2_ microspheres were used as the samples in this study. An aqueous (deionized water) suspension of uniform, non-porous (plain) SiO_2_ microspheres with a nominal diameter of 5.0 µm and a coefficient of variation of less than 15% (measured by the Coulter principle) was purchased from Bangs Laboratories (Fishers, IN, USA) (product code SS05003-1.0). The surface groups and densities of the non-porous SiO_2_ microspheres were Si-OH (non-functionalized) and 2.0 g/cm^3^, respectively. The non-porous SiO_2_ microsphere suspension was stored at 2–8 °C until use. Mesoporous SiO_2_ microspheres (SBA24 with a pore diameter of 23.5–23.6 nm) were synthesized based on previously reported methods [30,31]. Dried powder of the mesoporous SiO_2_ microspheres (SBA24) was stored at room temperature (20–25 °C) in a sealed desiccator until use. Dried powders of lactoferrin (LF) (product code 123-04124) and transferrin (TF) (product code 208-18971) were purchased from FUJIFILM Wako Pure Chemical Corporation (Osaka, Japan). The 10× phosphate-buffered saline (PBS) buffer (pH 7.4; product code 314-90185) and 10× Tris-buffered saline (TBS) buffer (pH 7.4; product code 317-90175) from Nippon Gene Corporation (Toyama, Japan) were diluted 10-fold with ultrapure water to prepare PBS and TBS, respectively. These solutions were then used to suspend the SiO_2_ microspheres.

For creating a calibration curve in spICP-MS, ion standard solutions of Si with different concentrations of 0–100 µg/L were prepared from 1000 mg/L single-element standard solution (Kanto Chemical Corporation, Tokyo, Japan).

### 3.2. Sample Preparation

Five microliters of an aqueous suspension containing approximately 1 mg of non-porous SiO_2_ microspheres was placed in a tube. An aqueous buffer (995 µL), PBS or TBS, was added to the tube and rigorously vortexed. The non-porous SiO_2_ microsphere suspension was diluted 20 times with buffer to a concentration of approximately 5 × 10^4^ particles/mL and used for subsequent experiments.

Approximately 1 mg of dry mesoporous SiO_2_ microspheres (SBA24) was weighed in a tube. One milliliter of an aqueous buffer, such as PBS or TBS, was added to the tube and rigorously vortexed twice for 3 s. For SiO_2_ equilibration, the resultant suspension was gently rotated at room temperature (20–25 °C) for 5 min. The mesoporous SiO_2_ microsphere suspension was diluted 400 times with buffer to a concentration of approximately 4 × 10^6^ particles/mL and used for subsequent experiments.

LF and TF were used as representative iron (Fe)-containing proteins to bind to mesoporous SiO_2_ microspheres (SBA24). Bottles of LF and TF stored in a refrigerator were left to stand for 15 to 30 min to return them to 20–25 °C. Approximately 1 mg of LF or TF was placed in each tube. One milliliter of TBS was added to the tube, gently vortexed for 3 s, and slowly rotated for 30 min at 20–25 °C for complete dissolution. Thereafter, the resultant solution was centrifuged at 19,000× *g* for 5 min at 20 °C. The supernatant (i.e., the dissolved protein fraction) was transferred to a new tube and used as a protein solution to prepare the protein-binding non-porous/mesoporous SiO_2_ microspheres.

### 3.3. Particle Size Measurement by spICP-MS

A quadrupole ICP-MS instrument (Agilent 7700x ICP-MS; Agilent Technologies, Santa Clara, CA, USA) equipped with an ICP torch with an injector tube of diameter 1.5 mm, a conventional MicroMist nebulizer, and a Scott double-pass spray chamber cooled at 2 °C was used for spICP-MS in combination with an externally assembled high-speed pulse signal processing system [32]. The ICP-MS instrument was tuned daily using a tuning solution containing 1 ng/mL each of Li, Co, Y, Ce, and Tl in 2% nitric acid to achieve optimum signal intensity and stability. The typical operating conditions of the ICP-MS instrument are listed in Table 1. Measurements were conducted in the helium (He) mode and at a dwell time of 100 µs. All samples were measured three times for a 60-s period each to ensure the detection of a sufficient number of particles; this enables the attainment of statistically reliable results. The cleaning time between samples with 2% nitric acid was 3 min.

Particle size measurements by spICP-MS were based on a conventional calibration approach using an ion standard solution (i.e., the ion standard solution approach) [33,34]. This approach uses a mass flux calibration curve from ion standard solutions and determines the particle size from the mass of the target particle, assuming a spherical geometry. Briefly, a calibration curve was constructed by relating the concentration of the ion standard solutions to the signal intensity. The concentration of the ion standard solution was then converted to mass flux using Equation (1):

(1) $$W=C_{STD}\times Q_{neb}\times t_{dwell}\times \eta$$

Here, *W* is the delivered mass per unit dwell time (ng), $C_{STD}$ is the mass concentration (ng/g), $Q_{neb}$ is the sample flow rate (g/s), $t_{dwell}$ is the dwell time (s), and η is the transport efficiency (%). The mass concentration, sample flow rate, dwell time, and transport efficiency were determined experimentally. The actual sample flow rate based on the nebulizer pump speed set at 0.10 rps was 0.352 g/min. Transport efficiency is defined as the ratio of the amount of analyte entering the ICP system to the amount of aspirated analyte. In this study, the particle-size method examined by Pace et al. [32] was applied to determine the transport efficiency. The signal intensity of each particle event was then substituted into the resulting mass–flux calibration curve with a slope of 1.4 × 10^8^ counts/µg, an intercept of 2.5 counts, and a correlation coefficient (*R*^2^) of 0.9990. The obtained signal intensities were converted to the masses of the corresponding particles using Equation (2),

(2) $$m_{P}=f^{-1}\times \frac{\left(I_{targetP}-I_{BKG}\right)}{m}$$

where $m_{P}$ is the mass of the particle, f is the mass fraction (the fraction of the particle mass due to the analyte element), $I_{targetP}$ is the signal intensity of the particle event, $I_{BKG}$ is the background signal intensity, and m is the slope of the mass–flux calibration curve. The resulting $m_{P}$ was converted to diameter ($D_{target\;P}$) using Equation (3), assuming a spherical geometry,

(3) $$D_{target\;P}=\sqrt[{3}]{\frac{6\times m_{P}}{\rho \times \pi \times \left(1-\varphi \right)}}$$

Here, ρ is the particle density and φ is the overall porosity (described below in detail). In the case of the non-porous SiO_2_ microspheres, the particle density (simply called ρ) was assumed to be equal to the density of the bulk material (2.65 g/cm^3^ for SiO_2_), similar to the assumption made in many previous studies [32,33,34]. In the case of the mesoporous SiO_2_ microsphere, the particle density ($\rho_{true}$) was measured as the “true density” using the gas pycnometry method following the procedure in the ISO 12154:2014 standard [35] and using a BELPycno helium pycnometer (MicrotracBEL, Osaka, Japana). The sample cell volume was 1 cm^3^, and the measurement temperature was set at 23 °C. Using the overall porosity (φ) and lower size detection limit for the non-porous (solid) particles (i.e., ${LOD}_{size,solid}$), the lower size detection limit for the porous particles (i.e., ${LOD}_{size,porous}$) can be calculated as follows.

(4) $${LOD}_{size,porous}=\frac{{LOD}_{size,solid}}{{\left(1-\varphi \right)}^{1/3}}$$

The value of ${LOD}_{size,solid}$ was determined using the method described by Lee et al. [23].

### 3.4. Porosity Determination

The overall porosity (φ) was determined for the mesoporous SiO_2_ microspheres using the following equation [36]:

(5) $$\varphi =\frac{V_{p}}{\frac{1}{\rho_{true}}+V_{p}}$$

Here, $V_{p}$ is the pore (void) volume and $\rho_{true}$ is the true density. The $V_{p}$ value was determined in-house using the nitrogen adsorption method [37].

Using the spICP-MS data and the average value of the particle diameters measured by SEM (explained in Section 3.5), the overall porosity (φ) of the microspheres was calculated using Equation (3). The calculated value was used only for discussion purposes.

### 3.5. Particle Size Measurement by SEM

A solution containing suspended SiO_2_ or MPS particles was dropped onto the carbon tape attached to the aluminum base, and excess water was removed using filter paper. This sample was dried for 5 min at room temperature (23 °C) and introduced into the FE-SEM (SU5000, Hitachi High-Tech Corp, Tokyo, Japan) instrument. Secondary electron images (1280 × 1020 pixels) were captured at 2000–2500× magnification with a scanning time of 20 s, a working distance of 7 mm, an EB acceleration voltage of 3–4 kV, and current of 1–5 pA. From 20 to 30 captured SEM images, 500 SiO_2_ and 400 MPS particle images were manually selected. The selected particle images were manually masked, and the diameter was calculated from the particle area using the masking region.

### 3.6. Detection of Protein-Binding Mesoporous SiO_2_ Microspheres by spICP-MS

An aqueous suspension (5 µL) containing approximately 1 mg of non-porous SiO_2_ microspheres, or approximately 1 mg of dry mesoporous SiO_2_ microspheres (SBA24) was placed in a tube. TBS (1 mL) was added to the tube and vortexed twice for 3 s. For SiO_2_ equilibration, the mesoporous SiO_2_ microsphere suspension was gently rotated at room temperature (20–25 °C) for 5 min. The solution in the tubes was then centrifuged at 19,000× *g* for 1 min at 20 °C. The supernatant was removed using a pipette tip. The prepared solution of Fe-containing proteins (i.e., LF or TF) was added to the tubes and rigorously vortexed twice for 3 s each time. For protein fixation, the solution was gently rotated at room temperature (20–25 °C) for 10 min. LF and TF bound to the non-porous/mesoporous SiO_2_ microspheres were detected by spICP-MS using Fe as a marker for the presence of proteins.

## 4. Conclusions

This study demonstrates that spICP-MS is applicable for particle size measurements and the detection of bound proteins in non-porous/mesoporous SiO_2_ microspheres.

In the future, we will expand the application areas of this technique. This would apply to larger non-porous/mesoporous particles if they can be fully decomposed by the plasma and their density and porosity are known. Moreover, it can also be applied to diverse types of mesoporous particles other than mesoporous SiO_2_ particles. For example, mesoporous TiO_2_ particles are widely recognized as photocatalysts and utilized in solar cells, lithium-ion batteries, biosensors, and cancer therapy [38,39]. Mesoporous Co_3_O_4_ particles have been exploited in the fields of energy storage, semiconductors, and catalysis [40,41]. The spICP-MS technique can be applied to the particle size measurement of mesoporous particles.

Further studies and applications of SiO_2_ particles will be of interest. Although nanometer-sized SiO_2_ particles (i.e., SiO_2_ NPs) have been highlighted in the literature, micrometer-sized SiO_2_ particles have unique features. For example, they seem to have more potential for medical applications than SiO_2_ NPs, as subcutaneously injected micrometer-sized particles have a higher decomposition speed in a living body [42]. The applications of micrometer-sized SiO_2_ particles can be enhanced by determining their particle sizes and detecting bound proteins using the spICP-MS technique.

## Figures and Tables

**Table 1:** Typical operating conditions of the ICP-MS instrument.

Parameter	Setting
Plasma and sampling conditions	
RF power	1550 W
Plasma gas flow rate	15 L/min
Auxiliary gas flow rate	0.90 L/min
Carrier (nebulizer) gas flow rate	0.90 L/min
Nebulizer pump	0.10 rps
Sampling position	10.0 mm
Cell gas (He) flow rate	3.0 mL/min
Data acquisition	
Scanning mode	Peak hopping
Data point per peak	1 point
Monitored isotope	^28^Si, ^57^Fe

## Data Availability

Data presented in this study are available upon request from the corresponding author. The data are not publicly available due to privacy concerns.
